# Physiologic response to distance diving in healthy children and young adults

**DOI:** 10.3389/fspor.2025.1515674

**Published:** 2025-03-28

**Authors:** Sasika Feige, Sophie Peter, Johannes Weickmann, Anna Michaelis, Roman Antonin Gebauer, Michael Weidenbach, Ingo Dähnert, David Münch, Max Poschart, Jan Wüstenfeld, Christian Paech

**Affiliations:** ^1^ Department for Pediatric Cardiology, University of Leipzig - Heart Center, Leipzig, Germany; ^2^ Head Office Finswimming, Landestauchsportverband Sachsen e.V., Leipzig, Germany; ^3^ Finswimming Department, SC DHfK Leipzig e.V., Leipzig, Germany; ^4^ Department of Sports Medicine, Charité – Universitätsmedizin Berlin, Corporate Member of Freie Universität Berlin and Humboldt Universität zu Berlin, Berlin, Germany; ^5^ Department of Sports Medicine, Institute for Applied Training Science (Institut für Angewandte Trainingswissenschaft (IAT)), Leipzig, Germany

**Keywords:** swimming, diving, children, pediatrics, adults, fin swimming, dynamic apnea, physiology

## Abstract

**Introduction:**

Diving and fin swimming are well established sports, including competitive sports, but little is known about the short-term adaptation of physiological parameters in Children and Adults during submersed dynamic apnea near the individual maximum. The current study provides data on the physiological adaptation of Children and young Adults to dynamic apnea diving in real—life conditions.

**Methods:**

This study provides data from 11 healthy elite fin swimmers (<30 years), including transcutaneous oxygen saturation and heart rate performing various diving protocols.

**Conclusion:**

The results suggest that apnea duration primarily affects oxygen saturation, while dive speed influences cardiovascular workload. Oxygen levels often decline post-dive, indicating a delayed oxygen debt requiring recovery. Future research should explore broader demographics, including recreational divers and medically restricted populations.

## 1 Introduction

Diving and fin swimming are well established-sports, including competitive sports, but little is known about the short-term adaptation of physiological parameters in Children and Adults during submersed dynamic apnea. Diving skills are vital in contact with open water and essential in the prevention of drowning accidents. Concerning the cardiovascular effects of immersion and submersion on the human body, patients with severe cardiopulmonary restrictions and congenital heart disease are partly restricted from taking part in swimming or diving activities to prevent potential harm based on physiologic assumptions (1, 2). Immersed in water, the human body has physiological mechanisms of adaption at its disposal to bridge the missing oxygen supply during apnea, which is most crucial to prevent blackout (3–5). The mammal diving response supports breath holding and leads to a reduced heart rate by activation of the vagal system (6). Peripheral vasoconstriction helps maintaining adequate blood flow to the brain and causes a centralization of blood to the oxygen sensible inner organs (7). Both mechanisms are already observable when breath holding with cold water face immersion (4, 8). Moreover, a reduction in spleen volume, caused by reflexive contraction, is observable not only in aquatic but in terrestrial mammals (9, 10). Due to hydrostatic pressure, full body water immersion increases stroke volume, reduces venous capacity, leads to restrictive ventilation and central hypervolemia causing renal diuresis (7, 11). Nevertheless, the human body seems to cope quite well with these external stressors, allowing to swim and dive to a remarkable extent (12). Previous studies of our working group justify the contemplation, that also cardiopulmonary restricted patients could possibly adapt quite well to this stressor (8, 13, 14). To assess expectable changes in transcutaneous O_2_ saturation (tO_2_%), heart rate (HR) and perfusion index (PI) during and following shallow dives in Children and young Adults and how these parameters behave when confronting the body with different diving times and workloads during the dive, this study provides data form elite fin swimmers in life-like conditions. As a consequence, this study may contribute to a more profound advice for athletes and patients who wish to safely participate in leisure or competitive swimming and apnea diving activities. We also wish to provide a data base for further considerations and research on this subject.

## 2 Methods

### 2.1 Participants' characteristics

Eleven healthy study subjects were recruited from local swimming and sports clubs. Inclusion criteria were young age (<30 years), experience with swimming and diving, i.e., participation in the regional fin swimming club, and the absence of cardiovascular disease in the patient history. Exclusion criteria were signs of reduced general condition and acute illness, signs of limitations of cardiopulmonary function, mental handicap and genetic diseases. Participants with common pre-existing respiratory conditions, such as mild allergies (two with house dust mite allergy and one with allergies to dog, cat, and grass pollen) or a history of bronchial hyperreactivity, were included, provided they were asymptomatic at the time of testing and showed no limitations in their physical activity. No signs of obstructive or restrictive ventilatory disorders were detected in any participants, and acute respiratory infections were excluded prior to testing. None of the measured blood parameters fell outside the normal reference values. The subjects or their legal guardians gave their informed consent. The study received ethical approval by the ethics committee University of Leipzig and is listed under the reference 034/24-ek.

### 2.2 Medical assessment and testing conditions

All study subjects underwent a thorough medical work up 14–20 days before the testing. Anthropometric data were measured. Cardiovascular and pulmonary data were collected by performing echocardiography (EPIQ CVx, Philips), electrocardiography (resting ECG, custo cardio 400, customed), and body plethysmography (Q-Box, CosMed). Blood samples were taken to assess hemoglobin levels and erythrocyte count, as these parameters are essential for oxygen transport. Data on potential medical history and medication was obtained from personal interviews. The testing took place in a local public swimming hall. Water temperature was 28°C and ambient temperature was 30°C. The pool was 2 m in depth. All dives were performed apnea and with monofins. The tests were supervised by a medical doctor and a study nurse.

### 2.3 Diving protocol

Prior to the start of the testing, the subjects were instructed to the following protocol. The protocol was designed in view of usual distances in diving competitions and in close consultation to the coaches of the fin swimming club, to warrant the study subjects safety on the one hand, and to set distances challenging enough for the study subjects to accomplish a dive near the personal maximum, on the other hand.

#### 2.3.1 Diving protocol for adults

Physiological data during and following a dive of different distance and different workloads was collected via pulse oximeter for an overall time of 70 min. The participants were asked to perform a 50, 75 and 100 m dive in a convenient, self-selected pace and two dives over 75 m of which one was economical pace and the other was at the study subjects individual maximum speed. Before and after, as well as between dives, monitoring in a sitting position for 10 min was applied. Mobile ultrasound measurement of spleen size in two diameters was performed prior to the first and after the last dive. An illustration of the test procedure for adults can be found in Supplementary Figure 1.

#### 2.3.2 Diving protocol for children (age 8–16)

Physiological data during and following a dive of different distances was collected via pulse oximeter for an overall time of 45 min. The participants were asked to perform a 25, 50 and 75 m dive in a convenient, self-selected pace. Before and after, as well as between dives, monitoring in a sitting position for 10 min was applied. Mobile ultrasound measurement of spleen size in two diameters was performed prior to the first and after the last dive. An illustration of the test procedure for children can be found in Supplementary Figure 2.

### 2.4 Assessment of physiological parameters during testing

Transcutaneous oxygen saturation, heart rate, and perfusion index (PI), as a surrogate of peripheral vascular tone (15, 16), were recorded for the whole testing period. PI is derived from the photoelectric plethysmography signal of the pulse oximeter and calculated as the ratio between the pulsatile component and the non-pulsatile component of the light reaching the detector. As the change in peripheral perfusion leads to a change in the pulsatile (arterial) component, whereas the non-pulsatile part, which is determined by the tissue, does not change, the ratio between both components changes (15). Thus, PI reflects peripheral vasomotor tone and is a surrogate for vasoconstriction or vasodilatation, promoting a volume shift. The perfusion index subsumes vascular tone and systemic blood flow, which is influenced by stroke volume (15).

Measurements were done using a Masimo Rad-97™ patient monitor and Masimo RD SET sensors (Masimo Corporation, Irvine, USA) protected from the water by hydrophobic coating with vaseline, which were placed on an index finger like demonstrated in the picture (Supplementary Image 1). The first mobile ultrasound measurement of spleen size was performed during the rest phase before the first dive, and the second measurement was performed after the last dive of the protocol, using a GE VScan with Dual Portable Ultrasound. Measurement was taken in transvers maximal length and maximal dorsoventral width, calculating spleen surface in cm^2^.

### 2.5 Statistical analysis

For the statistical analysis, IBM SPSS Statistics for Windows (V29) was used. Anthropometric measurements of the participants were analyzed and a Mann–Whitney *U* test was conducted to compare gender-specific differences within the study population. Differences in recovery time, oxygen and heart rate responses were assessed, with specific attention to variations between Adults and Children, as well as gender differences, using the Mann–Whitney *U* test. For the statistical analysis of the 75-meter dives, a Wilcoxon signed-rank test was employed to compare the physiological variables between the two different dive speeds. Spearman's correlation was applied to estimate the influence of dive time, speed and anthropometric data on oxygen and heart rate progression during the dives. The correlation coefficient (*ρ*) and *p*-value are included in the manuscript.

## 3 Results

The total study population of 11 fin swimmers was divided into a group of Children (<17 years) and a group of Adults (≥17 years). The characteristics of the study population are shown in Table 1. Children and Adults did not differ significantly in anthropometric as well as in cardiovascular and pulmonary data. There were significant differences between male and female patients. *P*-values for sex differences are shown in the last row of Table 1. Detected via mobile echocardiography, the mean reduction in spleen surface was 4.03%, indicating a notable adaptation over time.

**Table 1:** Characteristics of the study population.

Subjects characteristics	Group of children				Group of adults				Total study population		
	Female (*N* = 3)		Male (*N* = 2)		Female (*N* = 3)		Male (*N* = 3)		Female (*N* = 6)	Male (*N* = 5)	*p*-value
	Median	Range	Median	Range	Median	Range	Median	Range	Median	Median	
Age (years)	15	15–15	15.5	15–16	17	17–23	18	17–29	15–23	15–29	0.396
Anthropometry											
Size (cm)	162.3	161.9–167.2	180.9	178.4–183.4	169	166.7–170	176.9	170.2–192.7	161.9–170.0	170.2–192.7	0.006*
Weight (kg)	63.9	56.8–65.6	74.6	67.2–82.0	59.3	59.3–68.0	84.5	64.4–98.9	56.8–68.0	64.4–98.9	0.028*
BMI (kg/m^2^)	24.3	20.3–25.0	22.75	21.1–24.4	21.3	20.5–23.8	26.6	22.2–27.0	20.3–25.0	21.1–27.0	0.201
Body fat (%)	21.1	17.9–25.3	14.45	14.3–14.6	17.2	13.9–20.7	12.5	10.2–13.0	13.9–25.3	10.2–14.6	0.018*
Lean body mass (%)	49	46.6–50.4	63.8	57.6–70.0	51.1	49.1–53.9	75.9	56.4–86.0	46.6–53.9	56.4–86.0	0.006*
Haemogram											
Erythrocytes (Mio./µl)	4.62	3.89–4.86	4..97	4.97–4.97	4.85	4.60–5.00	4.84	4.76–5.42	3.89–5.00	4.76–5.42	0.200
Hemoglobin (mmol/L)	8.7	7.8–8.9	9.2	8.9–9.5	9	8.4–9.6	9.2	9.1–10.1	7.8–9.6	8.9–10.1	0.082
ECG											
QRS complex (ms)	91	86–96	109	101–117	103	102–109	106	99–118	86–109	99–118	0.144
Echocardiography											
Ejection fraction (%)	66.47	57.70–66.65	62.65	62.38–62.92	62.49	62.26–67.47	61.1	58.76–72.55	57.70–67.47	58.76–72.55	0.715
Absolute heart volume^a^ (ml)	613.7	540.47–706.48	888.06	866.71–909.40	637.83	587.74–696.33	948.4	743.62–1,098.34	540.473–706.475	743.624–1,098.340	0.006*
Relative heart volume^a^ (ml/kgKG)	9.60	9.52–10.77	11.99	11.09–12.90	10.24	9.91–10.76	11.2	11.11–11.55	9.52–10.77	11.09–12.90	0.006*
PFTs											
FEV1 (L)	3.08	2.56–3.17	4.59	4.19–4.99	3.93	3.36–3.97	4.82	3.86–5.49	2.56–3.97	3.86–5.49	0.018*
RV (L)	1.07	0.73–1.13	1.2	0.61–1.78	1.05	0.87–1.05	1.02	0.36–1.24	0.73–1.13	0.36–1.78	0.855
TLC (L)	4.96	4.81–5.29	6.96	5.49–8.42	5.51	5.04–5.95	7.57	5.41–7.85	4.81–5.95	5.41–8.42	0.045*
VC (L)	4.16	3.74–4.23	5.76	4.88–6.64	4.46	4.17–4.90	6.33	5.05–6.83	3.74–4.90	4.88–6.83	0.011*

^a^ Formula was based on the work of Dickhuth.

* Significant at a level of *α* = 0.05.

### 3.1 Physiological adaptation parameters during the dives

**Figure 1:**
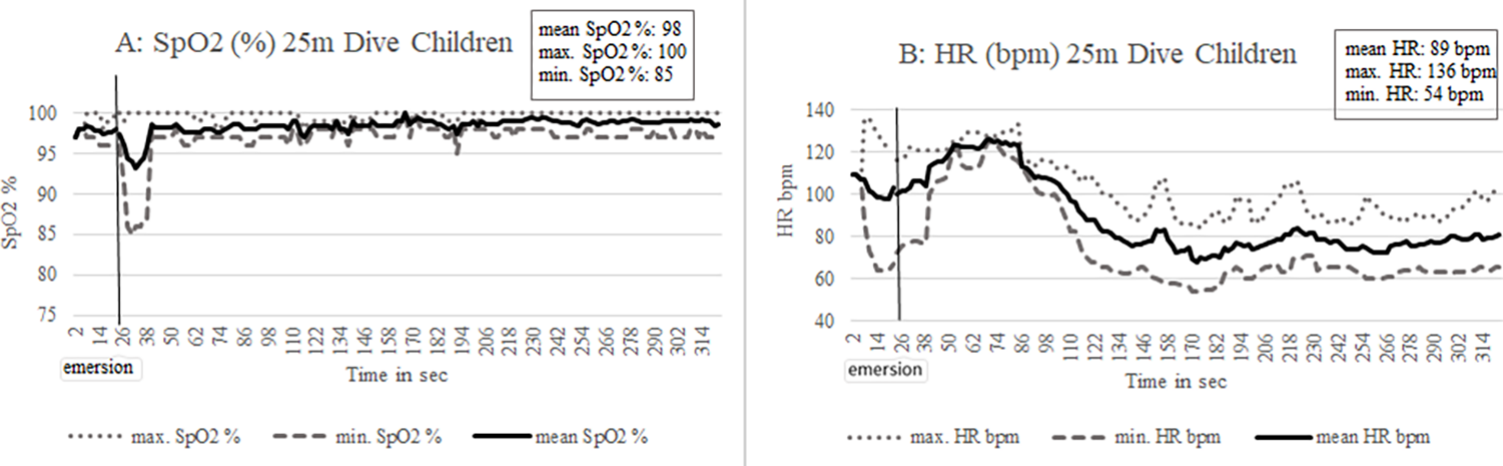
Twenty-five meters dive children: average, maximum and minimum values of transcutaneous oxygen saturation **(A)** and heart rate **(B)** are displayed over dive time (total average, maximum and minimum values are shown in the box).

**Figure 2:**
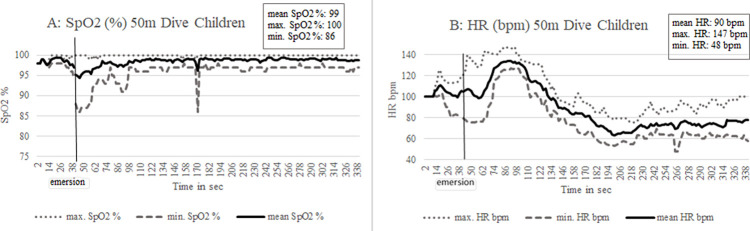
Fifty meters dive children: average, maximum and minimum values of transcutaneous oxygen saturation **(A)** and heart rate **(B)** are displayed over dive time (total average, maximum and minimum values are shown in the box).

**Figure 3:**
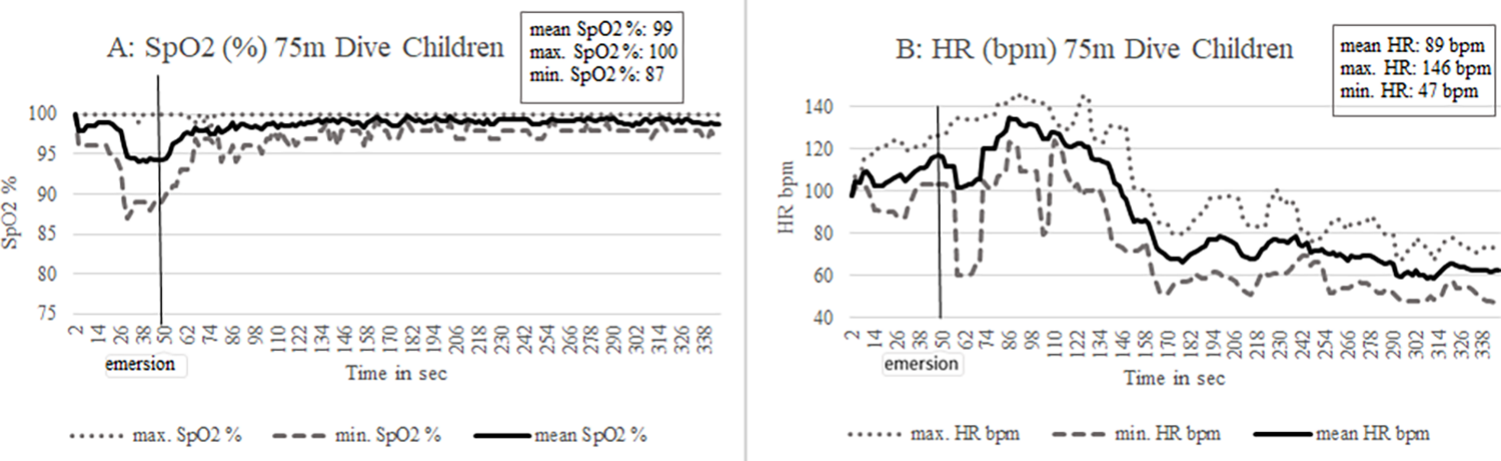
Seventy-five meters dive children: average, maximum and minimum values of transcutaneous oxygen saturation **(A)** and heart rate **(B)** are displayed over dive time (total average, maximum and minimum values are shown in the box).

**Figure 4:**
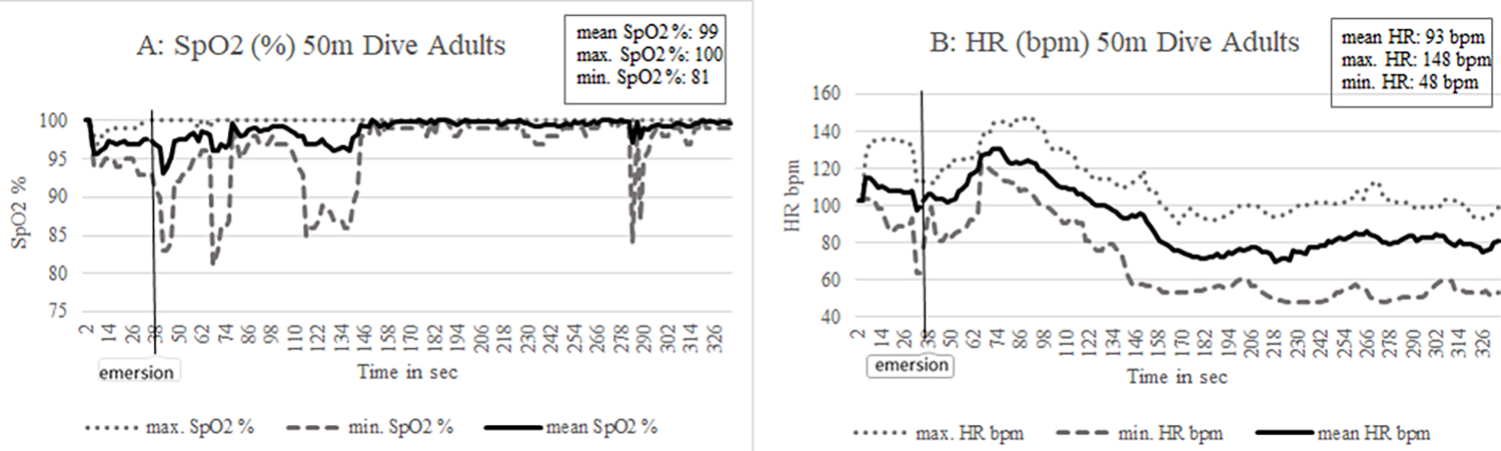
Fifty meters dive adults: average, maximum and minimum values of transcutaneous oxygen saturation **(A)** and heart rate **(B)** are displayed over dive time (total average, maximum and minimum values are shown in the box).

**Figure 5:**
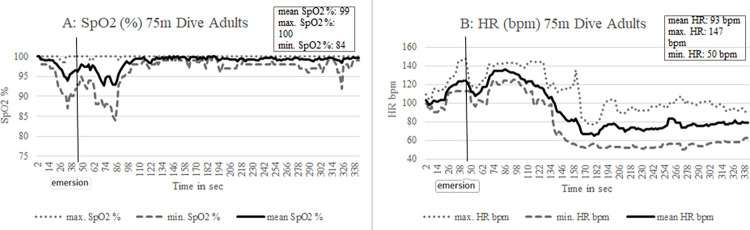
Seventy-five meters dive adults: average, maximum and minimum values of transcutaneous oxygen saturation **(A)** and heart rate **(B)** are displayed over dive time (total average, maximum and minimum values are shown in the box).

**Figure 6:**
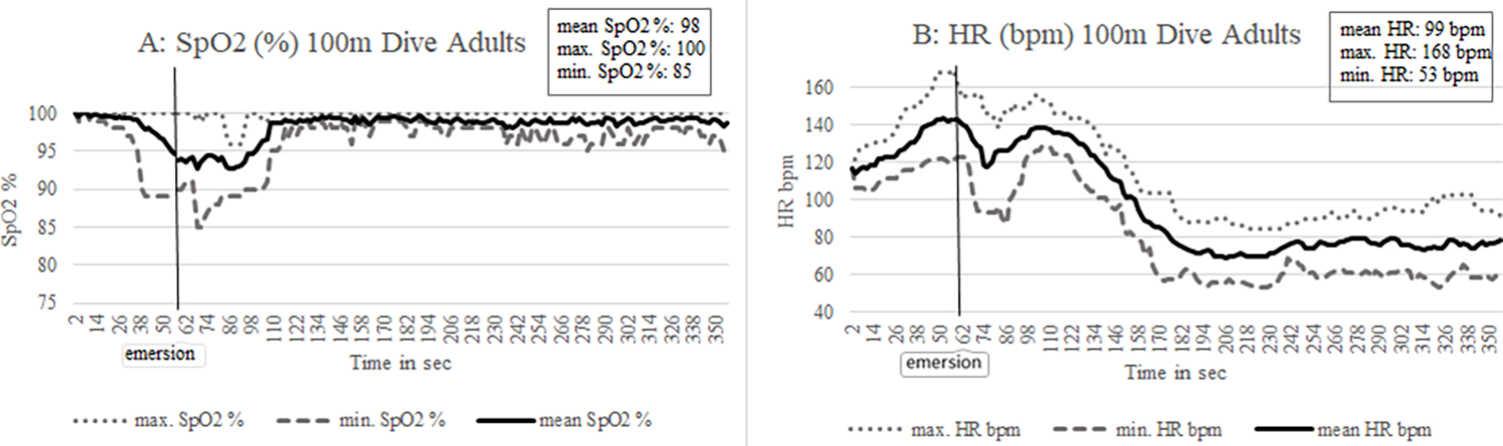
Hundred meters dive adults: average, maximum and minimum values of transcutaneous oxygen saturation **(A)** and heart rate **(B)** are displayed over dive time (total average, maximum and minimum values are shown in the box).

The dives over different distances were analyzed over time for heart rate and transcutaneous oxygen saturation separately with a 5 min follow-up period after emersion. Graphical representation for each distance and group is provided in Figures 1–6.

The time under immersion variated between the study subjects as the diving speed was individually determined. This was not taken into account in the diagrams (Figures 1–6) in favor of showing the phase directly after surfacing. Heart rate reduction following the immersion was not obligatory but most clearly observable during the 50 m dive with an average drop of 18 bpm (range 6–39 bpm; *N* = 6). While heart rate changes were evident in all other participants over 50 m, two Children and two Adults showed no change in heart rate. One adult individual even experienced an increase in heart rate by 10 bpm during the 50 m dive. Looking at the maximum heart rate of the 25–75 m dives, all participants reached their maximum heart rate only after surfacing when they had left the water, which is indicative of the diving bradycardia response. An exception was the longest dive distance in this protocol of 100 m. All participants experienced an average increase in heart rate of 24 bpm (range 14–37 bpm) during the underwater time. Four study subjects reached their maximum heart rate when emerging while 2 study subjects reached their peak after leaving the water. The lowest oxygen saturation values were measured after surfacing, although in average there was a slight drop in oxygen saturation levels during the longer dives. The curve of minimal oxygen saturation exhibited noticeable fluctuations, which can largely be attributed to the limited number of participants in this study. Given the small sample size, individual variability had a pronounced impact on the overall trend, as each participant's values were only recorded during a single event of desaturation. This overlap suggests that the desaturation phase occurred later in some participants than others. These factors should be considered when interpreting the results, and future studies with a larger cohort may provide a more consistent curve. One exception must be made for an adult participant, in which the lowest values were reached continuously during the dive (50 m: 95% SpO_2_; 75 m: 87% SpO_2_; 100 m 89% SpO_2_).

Children and Adults were expected to follow similar courses of changes in oxygen saturation and heart rate in response to the immersion. In this study, the group of Children differed significantly from the group of Adults in minimal oxygen saturation levels post diving (*p* = 0.019). There was a greater drop in oxygen saturation levels in the adult group after emersion, most noticeable over 75 m.

### 3.2 Factors influencing minimum oxygen saturation and heart rate

No sex differences in the investigated parameters could be found. An analysis of all dives indicates a significant connection between dive time and cardiac work load. Dive time correlates moderately with mean heart rate (Spearman's *ρ* = .491, *p* = .005) as well as with maximum heart rate (Spearman's *ρ* = .528, *p* = .002). The time under water does also correlate with the intraindividual range from highest to lowest heart rate measured while diving (Spearman's *ρ* = .473, *p* = .007). Consequently, a longer dive time is associated with a higher heart rate, indicating a rising activation of autonomic nervous system. No correlation was found between dive time and minimal transcutaneous oxygen saturation under water (Spearman's *ρ* = −.073, *p* = .696). The PI showed significant drop with immersion, but no significant trend in the PI can be identified during the dives based on the available data.

### 3.3 Time for recovery

There was a resting period of 10 min after each dive. Table 2 shows the time in seconds until average values of transcutaneous oxygen saturation and heart rate prior to dive are restored after emersion. No statistically significant difference in recovery times for 50 and 75 m dive was found between Children and Adults (*α* = 0.05). Average recovery times for oxygen saturation were below 1 min with a maximum of 72 s (75 m dive, Adults) to regain the base level. Normalization of the heart rate takes at a minimum 1 min, with average values around 100 s and maximum times of 160 s (50 m dive, Children). Dive time did not correlate significantly with minimum oxygen saturation or maximum heart rate post diving in Spearmańs correlation [min SpO_2_ post diving (%): *ρ* = .011, *p* = .956; max heartrate post diving (1/min): *ρ* = .244, *p* = .202]. Minimum oxygen saturation level reached after emersion strongly affects the time to recover to baseline levels (Spearman's *ρ* = −.744, *p* = .000). The time needed to recover baseline oxygen saturation is also correlated moderately with maximum heart rate during the dive (Spearman's *ρ* = .429, *p* = .023), whereas the time to recover baseline heart rate shows no significant correlation with any oxygen saturation parameters during or after the dive.

**Table 2:** Recovery times for oxygen saturation and heart rate in children and adults.

		Group of children (*N* = 5)			Group of adults (*N* = 6)			
	Diving distance	Mean	Min.	Max.	Mean	Min.	Max.	*p*-value
Time to normalize tO_2_% (s)	25 m	4.67 (*N* = 3)	0	14				
	50 m	22.00	0	64	26.00 (*N* = 5)	0	44	0.597
	75 m	18.40	0	40	44.00 (*N* = 4)	20	72	0.086
	100 m				44.00	16	68	
Time to normalize heart rate (s)	25 m	125 (*N* = 2)	94	156				
	50 m	115.60	90	160	103.4 (*N* = 5)	86	125	0.602
	75 m	94.00	60	116	104.75 (*N* = 4)	94	122	0.462
	100 m				104.33	86	120	

The ratio between body fat percentage and lean body mass influences the minimal oxygen saturation post diving as well as the time to normalize SpO_2_%. There was a negative correlation between lean body mass and minimal oxygen saturation post diving (Spearman's *ρ* = −.405, *p* = .027). Besides, lean body mass correlated with time to normalize SpO_2_% (Spearman's *ρ* = .492, *p* = .008). Body fat percentage behaved in exactly the opposite way, correlating negatively with time to normalize SpO_2_% (Spearman's *ρ* = −.504, *p* = .006) and positively with minimal oxygen saturation post diving (Spearman's *ρ* = .387, *p* = .035).

### 3.4 Influence of dive speed

**Figure 7:**
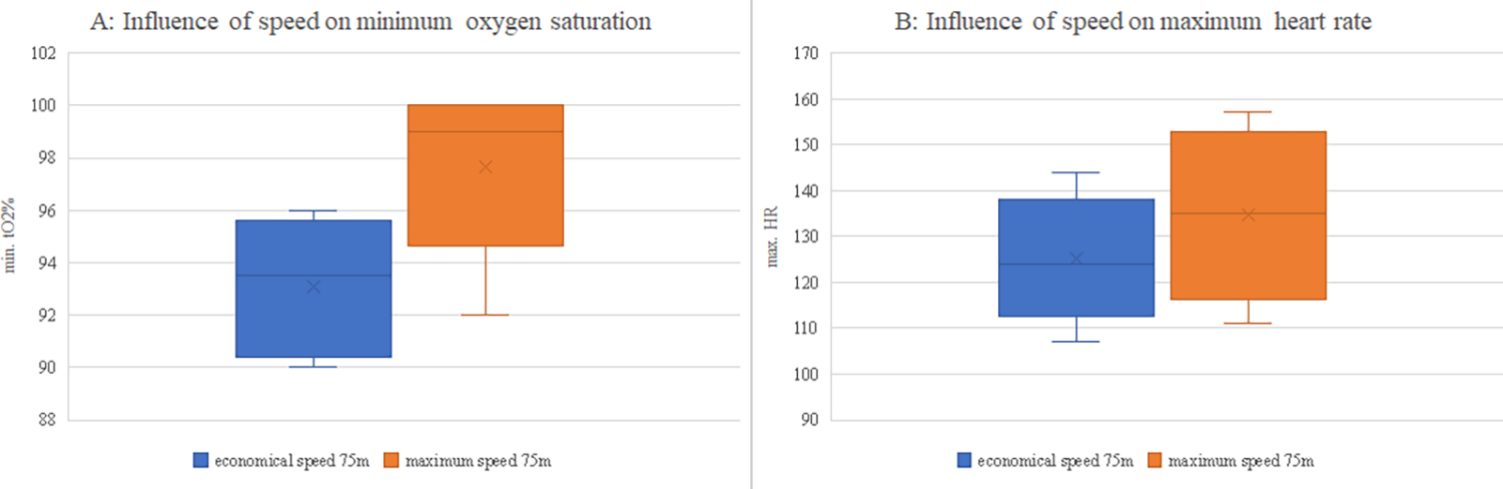
Comparison of minimum oxygen saturation **(A)** and maximum heart rate **(B)** between economic and maximum diving speeds.

Adult participants were asked to perform two 75-meter dives: one at an economical pace and the other at their individual maximum speed, to determine the influence of speed on oxygen saturation and heart rate. They adhered to the instruction and completed the second 75 m much faster than the first with an average pace of 1,96 m/s for the economical and 2,63 m/s for the maximum speed lap (*p* = 0.028). This had an impact on oxygen saturation and heart rate within the apnea phase as seen in Figure 7. Despite the expected lower muscular work load, a significantly larger drop in mean oxygen saturation (*p* = 0.028) and minimum oxygen saturation (*p* = 0.042) was seen while diving the individual more comfortable economical pace which automatically led to more apnea time. The cardiac response reflected the difference in muscular work load over the two dives. A dive with maximum speed, significantly increases mean heart rate (*p* = 0.028), minimum heart rate (*p* = 0.028) and maximum heart rate (*p* = 0.027) compared to a dive at an economical pace over the same distance. In total, the speed of the 75-meter dive was significantly correlated with the minimum heart rate underwater (Spearman's *ρ* = 0.669, *p* = .017), underlining the relationship between diving speed and cardiac workload. Interestingly, the recovery parameters seem to not differ significantly depending on the dive speed.

## 4 Discussion

This is the first study that examined the short-term physiological adaptations during dynamic apnea dives under life-like conditions. It provides a dataset of 11 elite fin swimming athletes, who were analyzed for heart rate, oxygen saturation, and perfusion index (PI) using transcutaneous pulse oximetry to better understand physiologic adaption parameters during dynamic apnea. The findings contribute important insights into the physiological demands of shallow apnea diving.

A central mechanism during apnea diving is the diving response (bradycardia and central volume shift by peripheral vasoconstriction), a phenomenon provoked by vagal influence (6). Our study corroborated this response in most subjects, with heart rate reductions observed after entering the water particularly over the 50 m dive. This aligns with other studies documenting diving-induced bradycardia in both Children and Adults (4, 8). The exception of increased heart rate over the 100 m dive with maximal heart rates reached already when emerging, most likely reflects sympathetic activation overpowering the parasympathetic trigger in autonomic regulation due to the heightened physical and metabolic stress and possibly psychological stress of longer dives (3). This assumption is underpinned by the moderate correlation found between dive duration and the intraindividual range from highest to lowest measured heart rate while diving. Interestingly, this phenomenon seems to be related to the work load and stress levels as no such correlation could be found between dive time and minimal oxygen saturation during the dive. The lowest oxygen saturation levels were reached after resurfacing, rather than during the dive itself. This supports the idea that the diving response—including bradycardia and peripheral vasoconstriction—helps conserve oxygen even under dynamic apnea (7). Once the dive ends and normal circulation resumes, the oxygen dept from peripheral tissues has to be paid off. This finding has practical implications for apnea training and safety protocols. Trainers and medical professionals should be aware that oxygen levels may not reach their lowest point during the dive itself, but rather just after it, making post-dive recovery observation, for at least 1 min, essential. Individuals with higher lean body mass showed lower post-diving SpO_2_% and delayed recovery in oxygen saturation levels. During a prolonged breath-hold, oxygen stores are gradually depleted and the body shifts toward anaerobic metabolism to sustain muscle function (17). Individuals with more lean body mass may accumulate higher levels of anaerobic byproducts like lactate and also have larger myoglobin for oxygen storage, which increases the oxygen debt that needs to be replenished after resurfacing.

Nevertheless, interindividual differences should be taken into account as in our small cohort there was one participant whose lowest oxygen values were reached continuously during the dive. No pre-disposing condition was found, that could explain these findings in this participant.

The protocol included both fast and slow dives over a distance of 75 m, allowing for a direct comparison of the impact of speed on heart rate and oxygen saturation. Faster dives resulted in greater cardiovascular stress, as seen in higher heart rates during maximum-speed dives. In contrast, a slower, subjectively more economical dive resulted in a more pronounced drop in oxygen saturation, as the apnea duration was extended. This suggests that oxygen reserves are primarily affected by the length of the apnea period rather than the intensity of physical effort when diving the same distance. On the other hand, faster dives are associated with a higher cardiac workload, as reflected by the significant increase in heart rate. Taking into account, that recovery parameters remain largely the same across both dive types, it highlights how different dive strategies can affect physiological responses. Based on specific goals it can be beneficial to either minimizing oxygen consumption or managing cardiovascular strain. While this study focused on the influence of speed over a fixed distance, it is worth investigating how speed affects physiological responses when dive duration is controlled instead of distance. By allowing participants to dive at varying speeds over the same time period, the interplay between speed, oxygen saturation, and heart rate might be more clearly delineated, offering a more comprehensive understanding of the effects of different diving strategies.

One main interest of this study was to find out if Children and Adults respond differently exposed to immersed dynamic apnea. Summarized, the investigated parameters of Children in comparison to Adults over 50 and 75 m behave very similar. Basic heart rate responses were equal. One exception must be made for the transcutaneous oxygen saturation levels after emersion, which were significantly lower in the adult group. Nevertheless, these findings have to be interpreted with caution, with the small number of study subjects in mind and with consideration for the fact that the two groups have similar cardiovascular and pulmonary data. Further research is needed to investigate differences in the diving response for dynamic apnea of Children and Adults with attention to the oxygen saturation levels after emersion. Interestingly, no sex differences in diving response were observable. There are expectable differences in anthropometric data as well as in cardiovascular and pulmonary data but these variations seem to have no general influence on the requested performance. Looking at world records stated by AIDA (International Association for the Development of Apnea) for static apnea, to date, 11:35 min are held by male and 09:07 min by female athletes (12). When it comes to peak performances, male subjects could have a slight physiological advantage as seen in many competitive sports. A comprehensive analysis of the study subjects characteristics shows a study population of moderate to highly trained subjects. Many participants of this study showed an incomplete right bundle branch block, indicating an increased vagal tone (18, 19). Former findings of studies on diving responses suggest a longer apnea time and more pronounced bradycardia reached by trained divers compared to untrained individuals (4). This allows the assumption, that the performance by trained fin swimmers seen in this study is above average which also means, that diving distance and apnea time would probably not be reached by merely recreational divers. Consequently, this dataset can be seen as an upper baseline, showing normal to above-average divers and their diving response and further studies are needed to investigate standard population and in the longer term even medically restricted subpopulations to complete the whole picture.

The diving response in mammals includes a reflexive contraction of the spleen whose significance still needs to be examined more closely (9, 10). Schagatay described a mean reduction of 18% in spleen volume after three apneas (9). Due to the test setting, we measured spleen surface in two diameters with mobile echocardiography. A slight reduction of 4% in surface area could be seen in our study population, underlining the interplay between immersion and spleen contraction already observed in former studies.

The analysis of the PI reveals no consistent trend. The authors consider multiple factors that may have contributed to these results, including contact with cold water by hand during the preparation phase and the limited measurement area. Other studies have documented a decrease in PI when the diving response is triggered by cold water face immersion due to the peripheral vasoconstriction (4, 8).

## 5 Conclusion

This study provides one of the first real life data sets reporting on physiological adaptations during dynamic apnea in the young, emphasizing significant distinctions between Children and Adults. Notably, while both Children and Adults exhibited similar heart rate responses, Adults experienced significantly lower post-dive oxygen saturation levels, emphasizing the need for further research into age-related differences in diving physiology. The results indicate that the duration of apnea primarily affects oxygen saturation, while dive speed influences cardiovascular workload. Interestingly, oxygen levels often decline only after the dive, suggesting a post-dive oxygen debt that must be addressed during recovery. This highlights the importance of post-dive recovery monitoring, as oxygen levels may continue to decrease after surfacing. This study lays a foundational dataset for understanding how the body adapts to the demands of dynamic apnea in Children and young Adults and future research should expand upon these findings by examining a broader demographic, including recreational divers and medically restricted populations.

## 6 Limitations

This prospective monocentric study was non-blinded, owed to the test setting and active surveillance of all participants while performing their protocol. The small number of 11 participants is the main limitation of this study. Data shows long diving periods with real-life locomotory muscle activity, but study subjects did not reach complete exhaustion. Thereby, we cannot predict critical cut-off values with certainty. A further limitation is the use of a clinical device not qualified for use under water. Although the pulse oximeter was well-prepared, we can not completely exclude aberrant values because of off-label use.

## Data Availability

The raw data supporting the conclusions of this article will be made available by the authors, without undue reservation.
