# Supplementary figures and images for: Physiologic response to distance diving in healthy children and young adults
Source: Front Sports Act Living. 2025 Mar 28;7:1515674. doi: 10.3389/fspor.2025.1515674 (PMC11985755; doi:10.3389/fspor.2025.1515674)

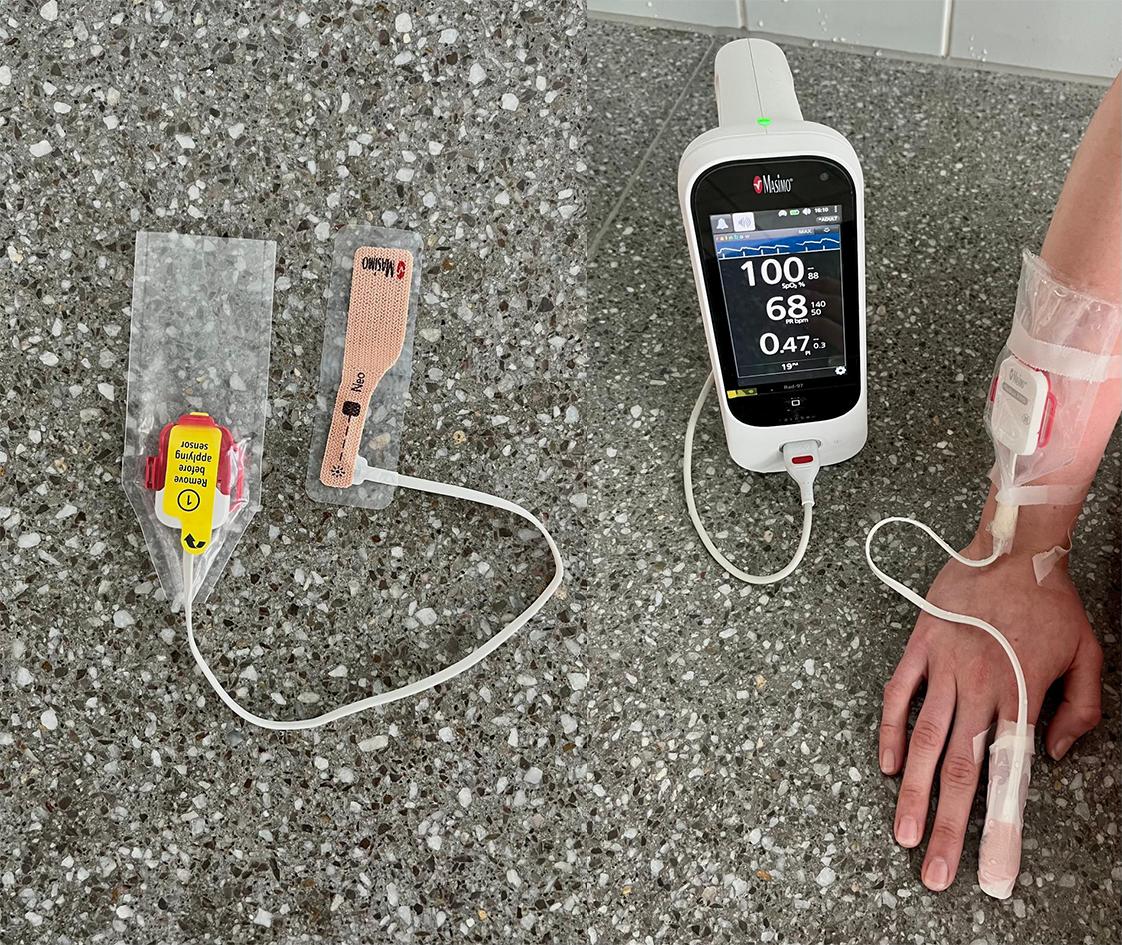

Supplement: Supplementary file 1 [file Image1.tif]

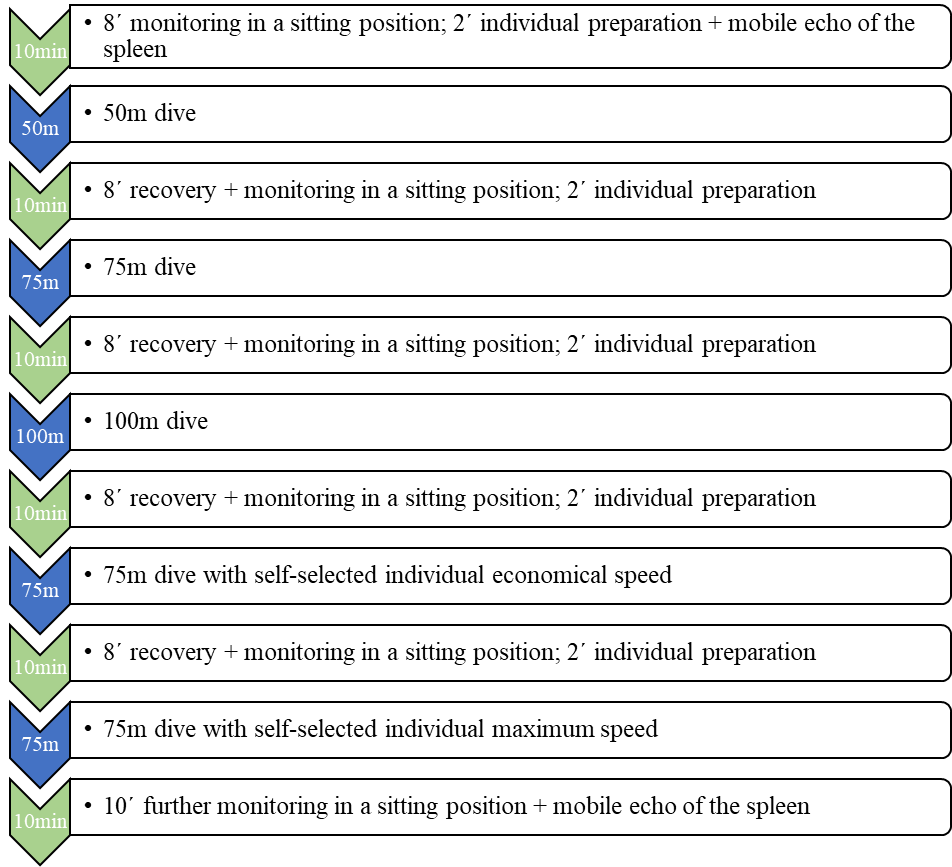


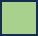
 ashore
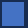
 under water

**Supplementary Figure 1:** Illustration of the test procedure for Adults

Supplement: Supplementary file 2 [file Table1.docx]

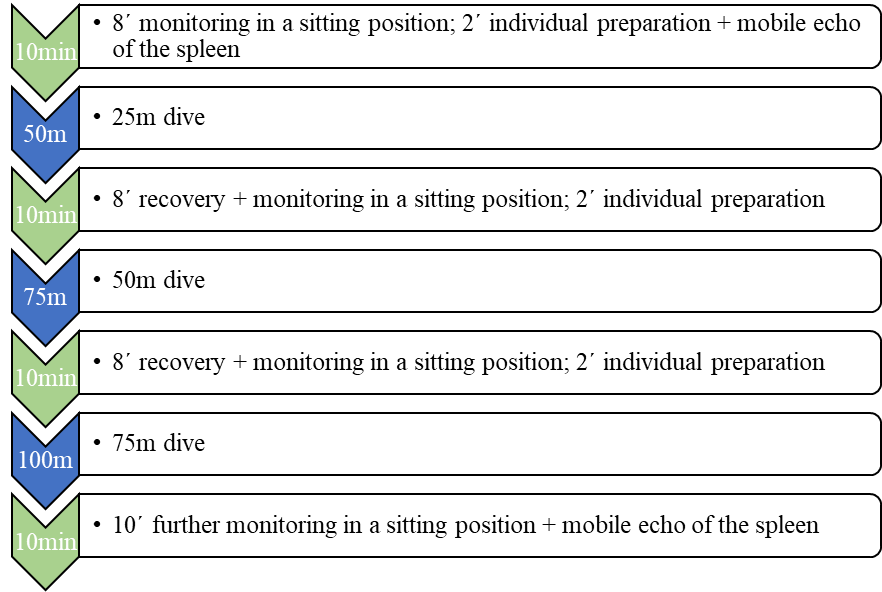


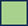
 ashore
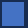
 under water

**Supplementary Figure 2:** Illustration of the test procedure for Children

Supplement: Supplementary file 3 [file Table2.docx]
